# Dual-Branch Convolutional Neural Network Based on Ultrasound Imaging in the Early Prediction of Neoadjuvant Chemotherapy Response in Patients With Locally Advanced Breast Cancer

**DOI:** 10.3389/fonc.2022.812463

**Published:** 2022-04-07

**Authors:** Jiang Xie, Huachan Shi, Chengrun Du, Xiangshuai Song, Jinzhu Wei, Qi Dong, Caifeng Wan

**Affiliations:** ^1^ School of Computer Engineering and Science, Shanghai University, Shanghai, China; ^2^ Department of Radiation Oncology, Fudan University Shanghai Cancer Center, Shanghai, China; ^3^ School of Medicine, Shanghai University, Shanghai, China; ^4^ Department of Ultrasound, Ren Ji Hospital, Shanghai Jiao Tong University School of Medicine, Shanghai, China

**Keywords:** deep learning, breast cancer, neoadjuvant chemotherapy, pathologic complete response, ultrasound imaging

## Abstract

The early prediction of a patient’s response to neoadjuvant chemotherapy (NAC) in breast cancer treatment is crucial for guiding therapy decisions. We aimed to develop a novel approach, named the dual-branch convolutional neural network (DBNN), based on deep learning that uses ultrasound (US) images for the early prediction of NAC response in patients with locally advanced breast cancer (LABC). This retrospective study included 114 women who were monitored with US during pretreatment (NAC _pre_) and after one cycle of NAC (NAC_1_). Pathologic complete response (pCR) was defined as no residual invasive carcinoma in the breast. For predicting pCR, the data were randomly split into a training set and test set (4:1). DBNN with US images was proposed to predict pCR early in breast cancer patients who received NAC. The connection between pretreatment data and data obtained after the first cycle of NAC was considered through the feature sharing of different branches. Moreover, the importance of data in various stages was emphasized by changing the weight of the two paths to classify those with pCR. The optimal model architecture of DBNN was determined by two ablation experiments. The diagnostic performance of DBNN for predicting pCR was compared with that of four methods from the latest research. To further validate the potential of DBNN in the early prediction of NAC response, the data from NAC _pre_ and NAC_1_ were separately assessed. In the prediction of pCR, the highest diagnostic performance was obtained when combining the US image information of NAC _pre_ and NAC_1_ (area under the receiver operating characteristic curve (AUC): 0.939; 95% confidence interval (CI): 0.907, 0.972; F1-score: 0.850; overall accuracy: 87.5%; sensitivity: 90.67%; and specificity: 85.67%), and the diagnostic performance with the combined data was superior to the performance when only NAC _pre_ (AUC: 0.730; 95% CI: 0.657, 0.802; F1-score: 0.675; sensitivity: 76.00%; and specificity: 68.38%) or NAC_1_ (AUC: 0.739; 95% CI: 0.664, 0.813; F1-score: 0.611; sensitivity: 53.33%; and specificity: 86.32%) (p<0.01) was used. As a noninvasive prediction tool, DBNN can achieve outstanding results in the early prediction of NAC response in patients with LABC when combining the US data of NAC _pre_ and NAC_1_.

## Introduction

Breast cancer is the most common cause of cancer-related death among women worldwide (1). Neoadjuvant chemotherapy (NAC) has been used as a systematic preoperative treatment for patients with locally advanced breast cancer (LABC) (2). NAC has the advantage of downsizing breast cancers, thus allowing breast-conserving surgery and assessments of the response to chemotherapy during treatment. The achievement of pathologic complete response (pCR) may be a potential independent predictor of better disease-free survival (DFS) and overall survival (OS), especially in patients with triple-negative and human epidermal growth factor 2 (HER2)-enriched breast cancer (3). However, even with the continuous improvements in chemotherapy regimens, the number of patients who achieve pCR remains low (4). Due to the different molecular types and histopathology of breast cancer, the response to chemotherapy may be different. Therefore, identifying patients with superior responses to NAC early has naturally become one of the current hotspots of study.

The optimal method for monitoring the response to NAC has not been established (5). Imaging examination can be used as one of the primary assessment methods. Magnetic resonance imaging (MRI), US, and positron emission tomography (PET)/computed tomography (CT) have been used as evaluation tools (5–7). However, imaging examinations have limitations when used clinically because image interpretation is mainly based on a radiologist’s visual assessment and is not standardized. Furthermore, MRI and PEC/CT are expensive, and PEC/CT is radioactive, making them impractical for frequent scans of patients receiving NAC. Among those methods, ultrasound (US) may become the primary monitoring tool due to its reusability, versatility, sensitivity, and safety.

With the continuous development of deep learning, computer-aided diagnosis (CAD) has become an important research topic, especially in breast cancer research. CAD research has involved the classification (8), segmentation (9), and detection (10) of breast tumours. Especially for classification tasks, which mainly focus on the differentiation of benign and malignant breast tumours, CAD has attracted increasing attention from researchers (11). Deep convolutional neural networks (CNNs) have been widely applied to many healthcare and medical imaging works, leading to state-of-the-art results (12–16). The classification operation procedure of a CNN is that an input image is fed into the CNN to learn essential features and save these parameters as weights and biases to classify images (17). Recently, with the help of deep learning methods, there have been several published studies for predicting breast cancer treatment responses based on PET/CT and MRI images (18–20). El Adoui M et al. introduced a two-branch CNN for the early prediction of breast cancer response to chemotherapy using DCE-MRI volumes acquired before and after chemotherapy (18). Braman N et al. developed a CNN for predicting pCR to HER2-targeted NAC with pretreatment DCE-MRI (19). Choi J H et al. used a CNN algorithm based on Alexnet to predict responses to NAC for advanced breast cancer using PET and MRI images (20). Those studies have shown that deep learning has emerged as a promising tool for breast cancer response prediction.

High-resolution breast US images contain rich texture and echo features that, when combined with deep learning techniques, may potentially be used to achieve a highly accurate and noninvasive NAC response detection method. At present, there are some studies about the use of CAD with US images for predicting the response of breast cancer to NAC (21–23). However, most of these studies focus on feature engineering work based on semiautomatic intermediate steps, and the technique is labour intensive and time consuming. The accuracy of a deep network has far exceeded that of a traditional machine learning method based on handcrafted features (8). However, in the learning process of existing deep learning models, the correlation and importance of the data during different chemotherapy courses have been ignored, and the characteristics of the data have not been well grasped. The purpose of our study is to construct a novel deep learning-based approach named the dual-branch convolutional neural network (DBNN) based on US images at different stages of chemotherapy for the early prediction of NAC in patients with LABC.

## Methods

### Study Participants

**Figure 1:**
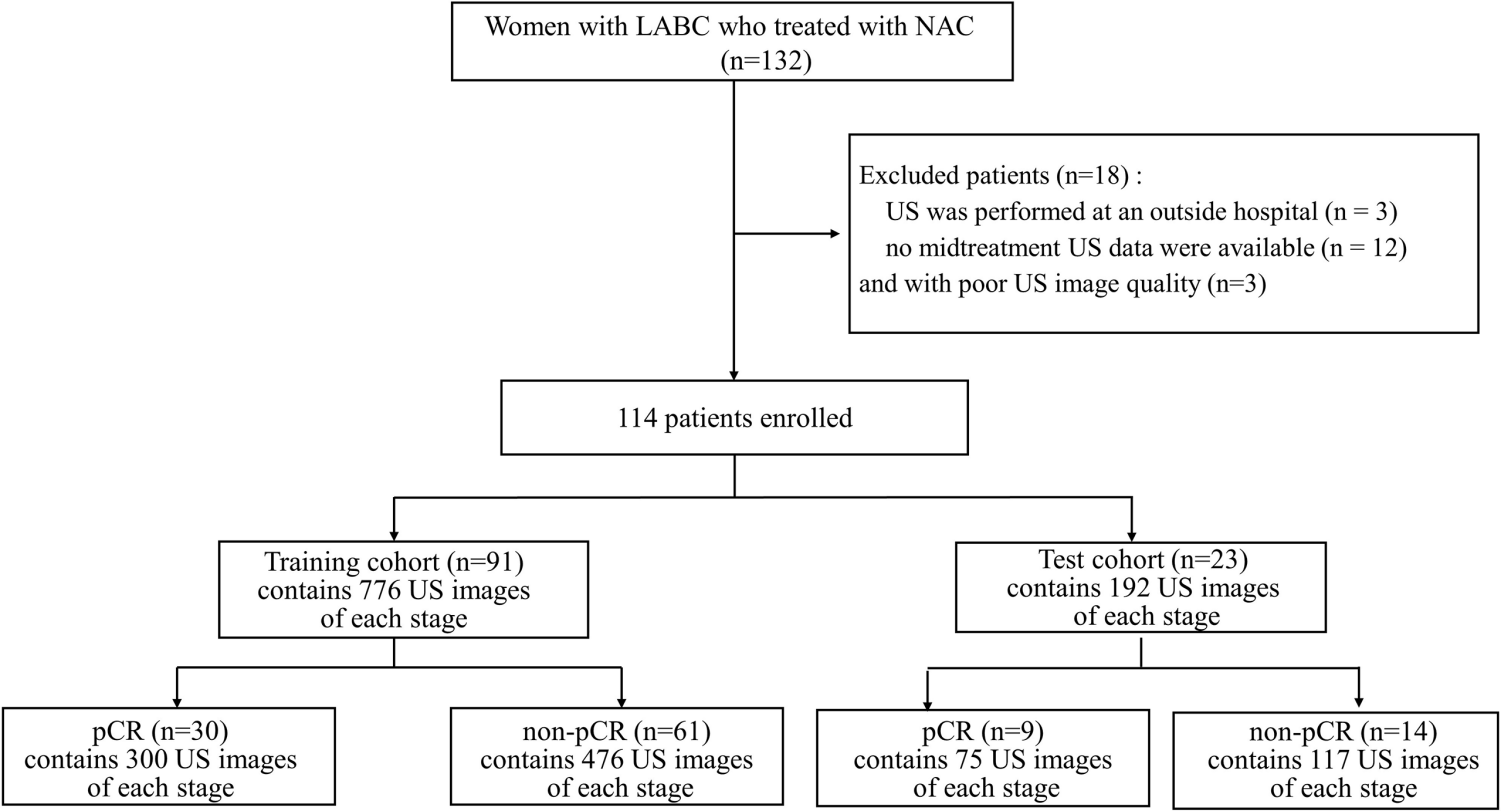
Flowchart for the study. LABC, Locally Advanced Breast Cancer; NAC, Neoadjuvant Chemotherapy; US, Ultrasound; pCR, Pathologic Complete Response; non-pCR, non-Pathologic Complete Response.

This retrospective single-centre study was approved by the Ethics Committee of ShangHai RenJi Hospital (ShangHai P.R. China), and the requirement for written informed consent was waived. Between February 2015 and June 2019, we enrolled 132 women with LABC who were treated with NAC and surgical resection at our institution. The eligibility criteria were as follows: (a) patients with breast cancer aged 18 to 80 years; (b) patients with histologically confirmed breast cancer and no history of treatment for breast cancer; (c) patients for which US was performed during NAC; and (d) after NAC, the patients underwent surgery and a pathological evaluation was performed. Of the 132 patients, 18 were excluded for the following reasons: (a) US was performed at an outside hospital (n= 3); (b) no midtreatment US data were available (n= 12); and (c) the US images were of poor quality (n=3). A total of 114 patients (age range: 26-72 years; mean age: 49.92 years) comprised the study group. (**Figure 1**).

### US Examination

**Figure 2:**
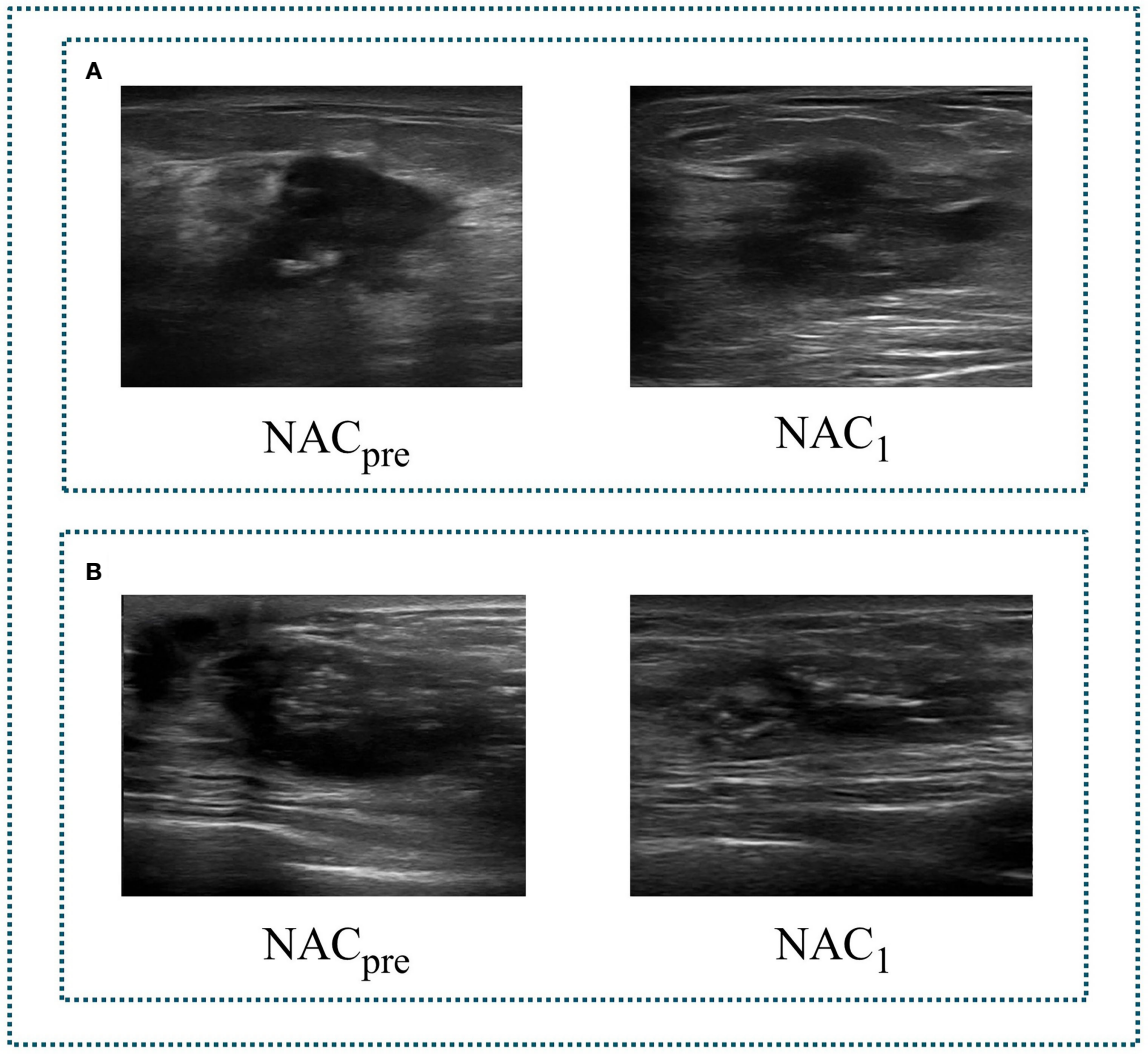
Two sets of tumour US images corresponding to different stages of NAC. **(A)** a set of images of pCR. **(B)** a set of images of non-pCR. NACpre, US images before chemotherapy; NAC1, US images after the first stage of chemotherapy.

The ultrasonography examinations were performed using MyLab Twice (Esaote, Genoa, Italy) with a 4–13-MHz LA523 linear transducer by an experienced radiologist at the Department of Ultrasound (C.F.W. with 10 years of experience in breast US). In this study, US images were collected before and after the first course of chemotherapy. The US images of pCR and non-pCR samples collected at different treatment stages are shown in **Figure 2**. The primary dataset called Renji NAC (RJNAC) contains 1936 (968×2 stages) US images (800×608 pixels) at different treatment stages, including 968 US images at each stage, with an average of 16 to 20 images per patient. For the prediction of pCR, the dataset was randomly split into training data (80%) and test data (20%) (a ratio of 4:1). That is, when dividing the dataset, the pCR and non-pCR ratios in the samples were kept close. In the training set and the test set, the pCR and non-pCR ratios were both approximately 0.63. Specifically, each stage of the training set contained 776 images, including 300 pCR images and 476 non-pCR images, while the test set contained 192 images, including 75 pCR images and 117 non-pCR images. (**Figure 1**).

### Data Preprocessing

**Figure 3:**
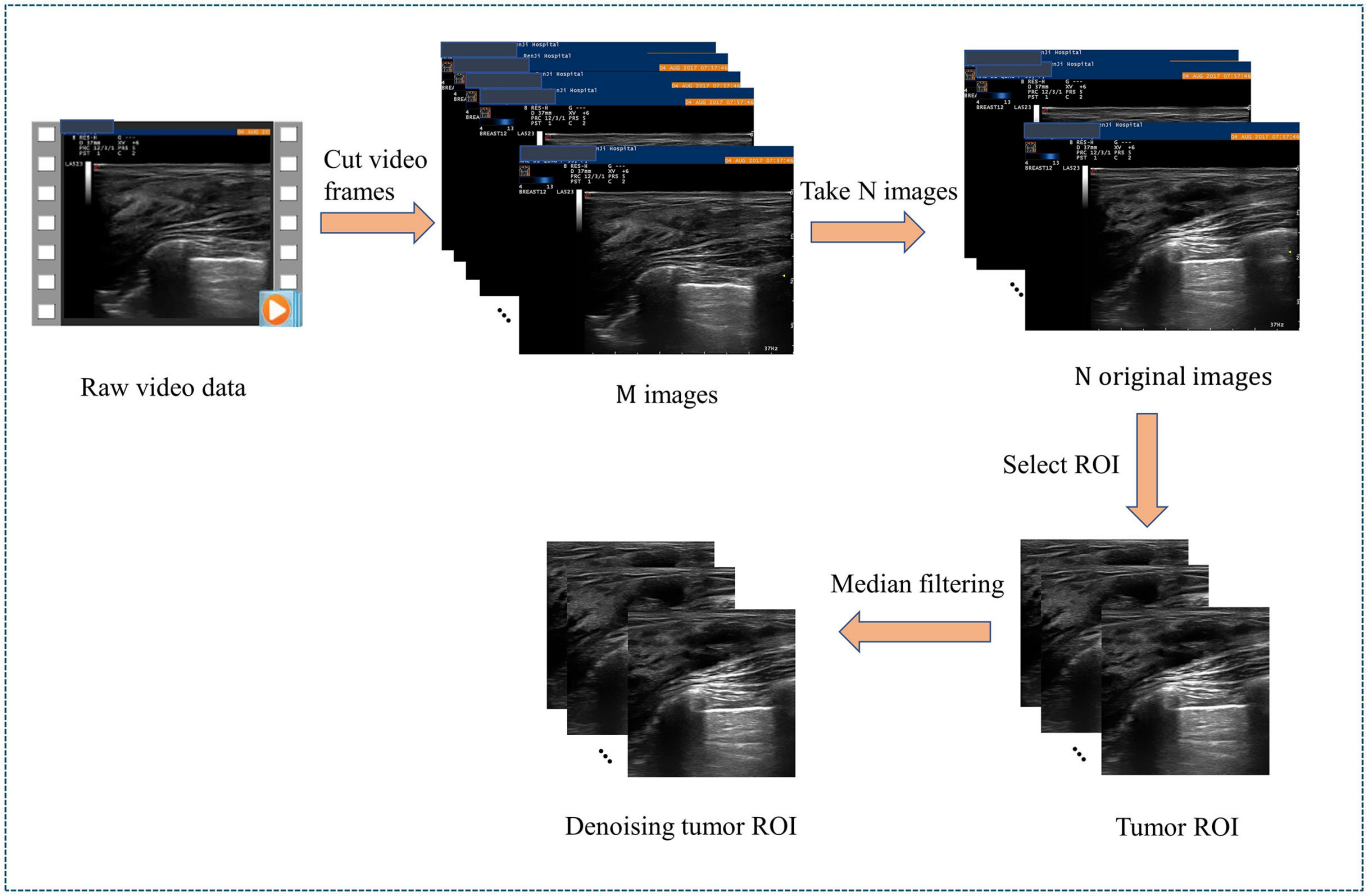
Data preprocessing of an ultrasonic video. ROI, Region Of Interest.

The data collected in this study are ultrasonic video data. To input it into the neural network, we perform a video frame cutting operation on the video data (24–26). Four preprocessing steps are applied before starting the training process. As detailed in **Figure 3**, the first step is to cut the video with different time lengths according to the fixed frame interval to form an indefinite number of M ultrasonic images. The second step is to select N high-quality breast tissue images by removing some images containing artifacts, blur, and non-lesion tissue. Blind to the patients’ private information and pathological results, two professional radiologists (Q.D. and C.F.W. with five and ten years of experience in breast US, respectively) independently read the breast US images. They reach a consensus through discussion to ensure the correctness and repeatability of the dataset. The N of two stages of each patient must be the same but can vary for different patients, depending on how many clear and usable mass images were contained in the indefinite number of M images of different patients. The change of N among different patients does not affect the model learning. N images of two stages are paired sequentially to ensure that the image pairs of each pair are closest in the video time sequence. The third step is that, after removing the nonrelevant breast tissue information, such as the model number of the instruments, time of scanning or imaging, and patient information, we retain the remaining information as a region of interest (ROI). In addition, the resolution of ROI images obtained after video processing is consistent with the resolution of ROI images obtained by static single frame cropping, both of which are 445×445 pixels. Finally, we use the median filter (27) to denoise the US images and preserve edge information. All US images are represented as greyscale images with sizes of 128 × 128 before being fed into the deep neural network.

### Dual-Branch Convolutional Neural Network

In the prediction of NAC response, the existing studies failed to take advantage of the correlation among multistage data and the importance of data at each chemotherapy stage (5, 28–30). To solve this problem, we developed a model named DBNN based on feature sharing and weight assignment to predict chemotherapy response by utilizing US images before and after the first stage of chemotherapy (NAC_pre_ and NAC_1_, respectively). Dual branches were designed to extract data features from NAC_pre_ and NAC_1_. There are feature-sharing modules between different branches so that the model could fully use the correlation of the data from each stage. In addition, the model has a weight assignment module, which considers the importance of different branch features and provides prior knowledge for accurate classification.

**Figure 4:**
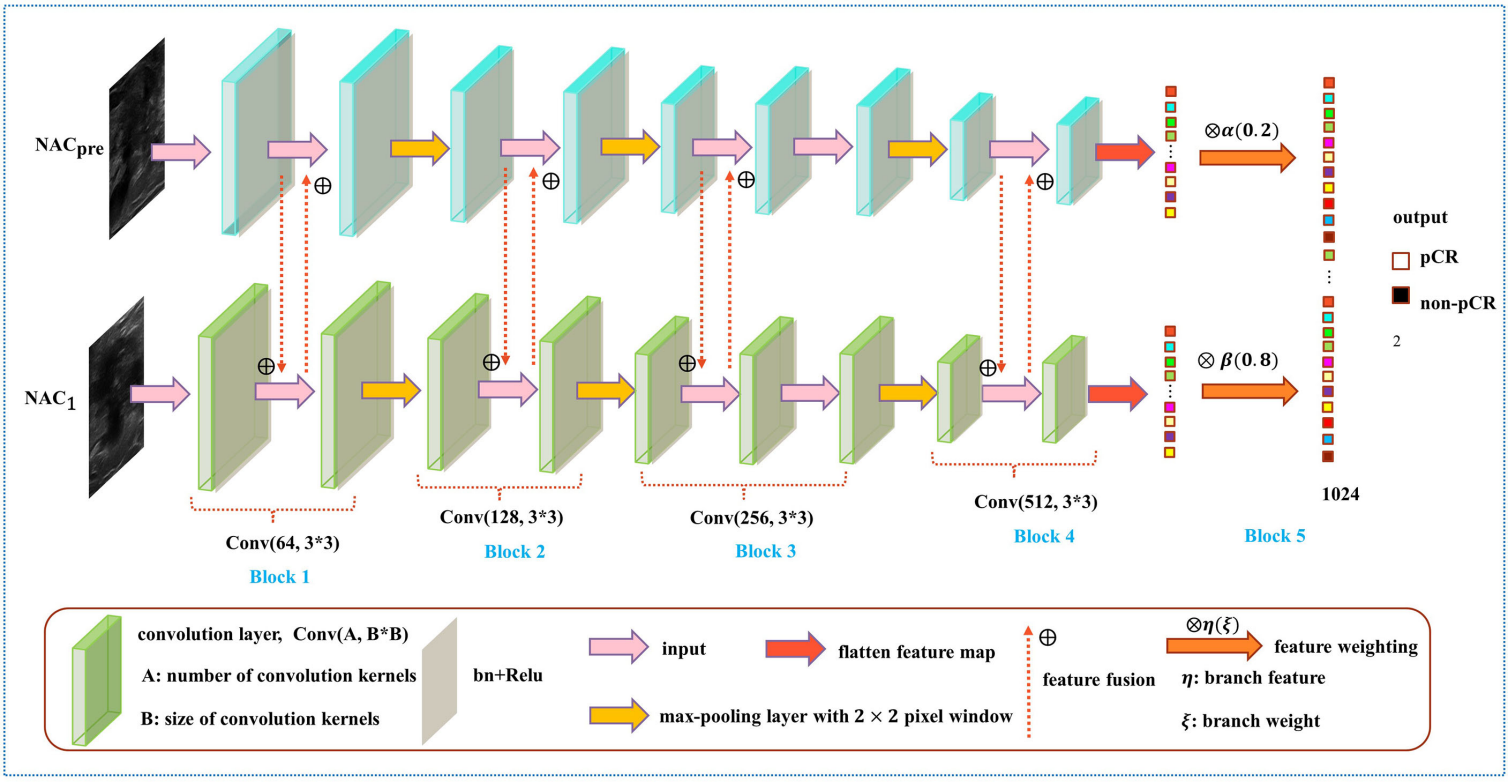
Overview of the DBNN model architecture.

As shown in **Figure 4**, the DBNN architecture is composed of two branches that take a 128 × 128 breast tumour ROI cropped from NAC_pre_ and NAC_1_ images as input. Each path contains four convolution blocks, which contain nine convolutional layers in total. Batch normalization layers (31) follow each convolutional layer to speed up network convergence, and a rectified linear unit (ReLU) activation function (32) is used to increase the nonlinearity of the network. Then, these layers are followed by four max-pooling layers (33), where each max-pooling layer is used to perform image downsampling. Furthermore, DBNN has two fully connected layers for feature weighting, and features are shared between each branch by feature fusion.

**Figure 5:**
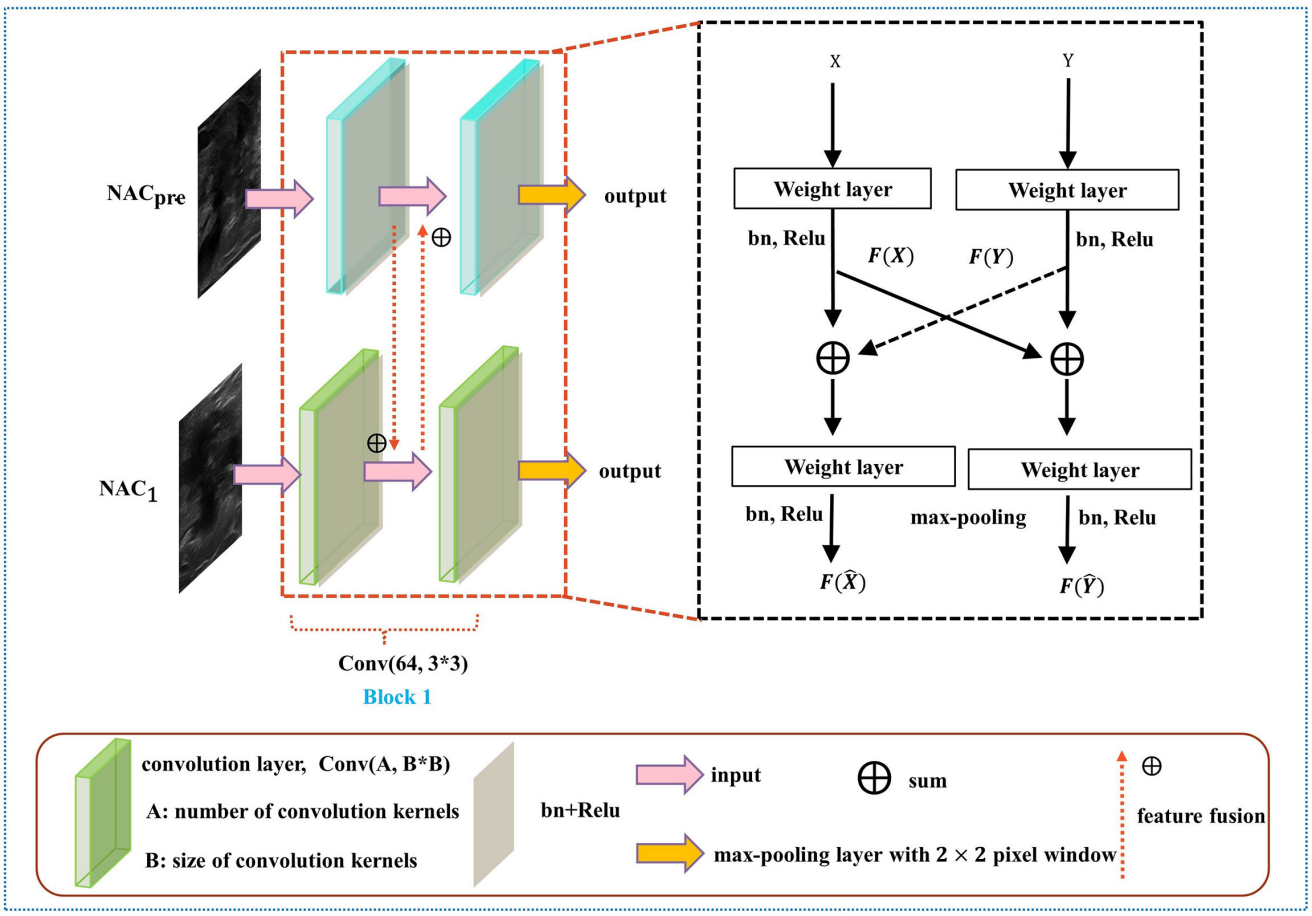
Diagram of the feature-sharing method.

The details of DBNN feature sharing are shown in the black dotted box in **Figure 5**. DBNN consists of four convolutional blocks, and the input of each block is the output of the previous block (except for Block 1, where the input is US images from NAC_pre_ and NAC_1_). Sixty-four kernels are used for each convolutional layer in Block 1, 128 for each layer in Block 2, 256 for each layer in Block 3 and 512 for each layer in Block 4, and each kernel has a size of 3 × 3. An US image is input into the respective branch at each stage. Then, the fusion feature map is trained through the convolutional layer, batch normalization layer, and ReLU function and finally downsampled and input into the other blocks until the convolution operation is completed.

First, the network starts from the input layer and is expressed as:


(1) $$C_{0}=X$$

(2) $$C_{0}^{'}=Y$$

where X denotes the input of NAC_pre_ and Y denotes the input of NAC_1_. Then, *C_0_* and $C_{0}^{'}$ are input to their respective convolution layers, and features are extracted through the convolution kernel. Finally, the feature maps *C_1_* and $C_{1}^{'}$ are generated. The formula is expressed as:


(3) $$C_{i}=\sigma_{i}\left(\omega_{i}\;*\;C_{i-1}+b_{i}\right)$$

(4) $$C_{i}^{'}=\sigma_{i}^{'}\left(\omega_{i}^{'}\;*\;C_{i-1}^{'}+b_{i}^{'}\right)$$

where *C_i_* and $C_{i}^{'}$ represent the feature maps of layer *i*, *i* ϵ{1,3,5,7,8}. *σ_i_* and $\sigma_{i}^{'}$ indicate the ReLU activation function, *ω_i_* and $\omega_{i}^{'}$ stand for the network weights of layer *i* of the two paths, *b_i_* and $b_{i}^{'}$ are network biases for the convolution layer, and * denotes the convolution operation. *C_i-_*_1_ and $C_{i-1}^{'}$ are used as inputs of the next layers, *C_i_* and $C_{i}^{'}$ , respectively.


(5) $$C_{j}=\sigma_{j}\left(\omega_{j}*\left(C_{j-1}+C_{j-1}^{'}\right)+b_{j}\right)$$

(6) $$C_{j}^{'}=\sigma_{j}^{'}\left(\omega_{j}^{'}*\left(C_{j-1}^{'}+C_{j-1}\right)+b_{j}^{'}\right)$$

where *C_j_* and $C_{j}^{'}$ represent the feature maps of layer *j*, *j* ϵ{2,4,6,9}. *C_j-_*_1_ and $C_{j-1}^{'}$ are used as inputs of the next layers, *C_j_* and $C_{j}^{'}$ , respectively.

After each convolution block, we obtain *C_k_* and $C_{k}^{'}$ and input them into the max-pooling layer to reduce the number of parameters of the feature map:


(7) $$C_{k}=maxpooling\left(C_{k}\right)$$

(8) $$C_{k}^{'}=\;maxpooling\left(C_{k}^{'}\right)$$

where *C_k_* and $C_{k}^{'}$ represent the feature maps of layer *k*, *k* ϵ{2,4,7,9}.

In contrast to the fusion method in the fully connected layer, DBNN shares the features between each branch; that is, it uses fusion when extracting low-level features. As a result, the model could be trained effectively to screen out crucial features, including changes in lesion areas before and after NAC treatment, thus affecting the prediction results of chemotherapy response.

**Figure 6:**
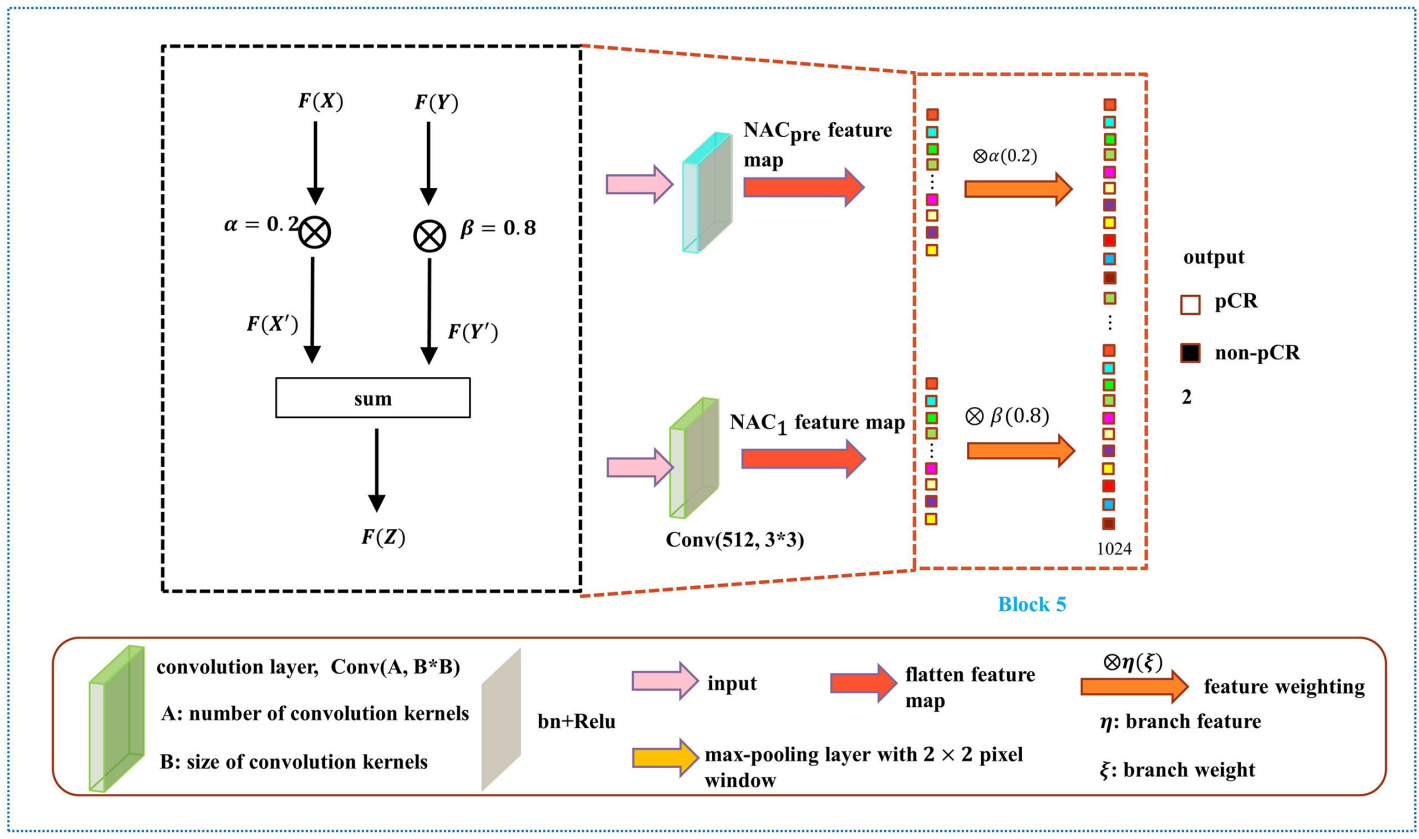
Diagram of the weight assignment method.

As shown in **Figure 6**, the weight fusion strategy of DBNN is uncomplicated, and the black dotted box shows the details of the red dotted box. First, the feature vector *F*(*X*) from the NAC_pre_ branch and the feature vector *F*(*Y*) from the NAC_1_ branch are input, and then the updated feature vectors *F*(*X'*) and *F*(*Y'*) are obtained by multiplying the two feature vectors by *α*(0.2) and *β*(0.8), respectively. Finally, the sum operation is performed on the updated features to obtain the feature vector *F*(*Z*) which is fused with the two branches. The process is expressed by the formula:


(9) F(Z)=(α ∗ F(X')+β ∗ F(Y'))

After the fully connected layer, we used a dropout strategy (34) (with a rate of 0.5), which helps to prevent the model from overfitting during training. Then, the two branches were summed after the fully connected layer with 1024 hidden units, and a softmax function was applied for pCR classification.

**Figure 7:**
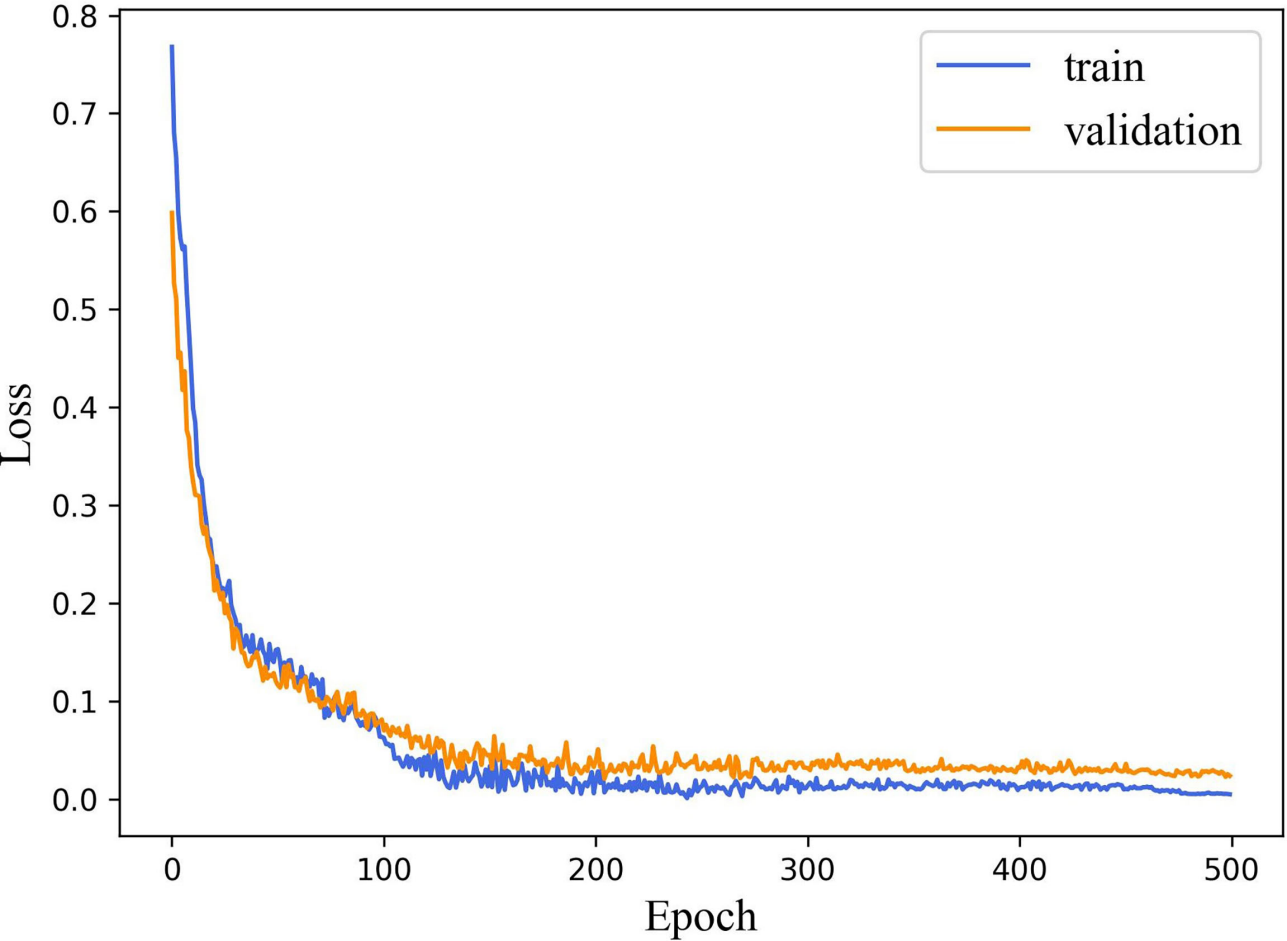
The loss curves of DBNN.

The performance of machine learning algorithms is primarily affected by their hyperparameters because their performance will be inferior without optimal hyperparameter values (35). In particular, the deep learning model relies on good hyperparameter values to accelerate the convergence of the model and achieve optimal performance. To compile and evaluate each model, we use cross entropy (36) as the loss function and a standard accuracy metric that calculates the mean accuracy rate across all predictions. **Table 1** shows the hyperparameter setup. The loss curves show no overfitting or underfitting in our model (**Figure 7**).

**Table 1:** The hyperparameters of the DBNN architecture.

Hyperparameter	Value
Optimizer	Adam (37)
Learning rate	0.001
Loss function	Cross entropy
Batch size	8
Epochs	500

All experiments were performed on a Dell T640 tower server deep learning workstation with two NVIDIA GeForce RTX 2080Ti independent graphics cards and two Intel Xeon Silver 4110 CPUs, with RAM extended to 64 GB. The experimental platform was in Python version 3.7. DBNN was implemented by PyTorch, which is a deep learning platform.

### Histopathologic Assessment

A pathologist with more than 20 years of experience in breast pathology assessed the histologic results. All pathologic results from outside biopsies were reviewed at our institution. Tumour pathologic characteristics were obtained from histopathologic reports of US guided core biopsies performed before NAC. The histologic type, grade, and expressions of HER2, the oestrogen receptor (ER), the progesterone receptor (PR), and antigen Ki67 were assessed. Tumours with >1% nuclear staining were denoted as ER/PR positive. The cut-off point for Ki-67 high expression was 30%. In terms of HER2 expression, tumours were considered HER2 negative if they had a score of 0 or 1+ during the immunohistochemical (IHC) examination, and a score of 3+ indicated that the tumour was HER2-positive. If the HER2 status was equivocal (IHC score: 2+ or 1+ to 2+), further investigation using *in situ* hybridization (ISH) was required. In our study, pCR was defined as no residual invasive carcinoma in the breast at surgical resection. Molecular subtypes were classified according to the St. Gallen Consensus (38).

### Statistical Analysis

Our statistical analysis was performed using IBM SPSS Statistics 22 (Armonk, NY, USA). Clinicopathological characteristics and US images before and after the first stage of chemotherapy, including maximum tumour diameter and tumour histologic type, were collected. The continuous variables were described as the range, mean and standard deviation, while the categorical variables were reported as counts with percentages. T-tests, chi-squared tests, or Fisher exact tests for independent samples were used to determine significant differences between the pCR and non-pCR groups. To evaluate the performance of the developed models, we calculated six performance metrics: accuracy, sensitivity, specificity, positive predictive value (PPV), negative predictive value (NPV), and F1-score. The predicted performance was assessed by using receiver operating characteristic (ROC) curves, and the area under the curve (AUC) scores were compared. Then, the results were analysed to select the best model to predict NAC response in patients with breast cancer utilizing breast US images. P <.05 was considered to indicate a significant difference. The performance results of the model and other methods were compared by using the Mann-Whitney U test. The 95% CIs for AUC were estimated by using the DeLong method (39–41). Statistical computing was implemented with the Scipy package, a Python-based open-source data processing tool. For the prediction of pCR, DBNN was trained on the training set and then validated on the test set.

F1-score conveys the balance between PPV and sensitivity. The closer the value is to 1, the better the performance of the method. The F1-score equation is defined as follows:


(10) $$F1-score=\frac{2TP}{2TP+FP+FN}$$

## Results

### Patient Characteristics

One hundred and fourteen women comprised the final study group (age range: 26-72 years; mean age: 49.92 years). The median maximum diameter of the tumours in the pretreatment US images was 3.82 cm (range: 1.35-8.2cm). The patient characteristics and the sizes of the tumours in the pCR and non-pCR groups are listed in **Table 2**. Of the 114 patients, 39 (34.2%) achieved pCR at the final pathologic evaluation. No significant differences were found in age, molecular subtype, or maximum tumour diameter between the pCR and non-pCR groups. For the 39 patients who achieved pCR, no residual invasive carcinoma in the breast or axillary lymph nodes was found in 37 (94.87%) patients. Thirty-seven (85.29%) patients showed no evidence of malignant cells in the breast, and 2 (8.82%) patients showed only ductal carcinoma in situ. Of the 75 patients with non-pCR, partial response was observed in 72 patients, and disease stability was observed in 3 patients. Disease progression was not observed for any patient in this study cohort. The pCR group showed a higher proportion of ER negativity (20 [51.3%], P <0.001), PR negativity (13 [33.3%], P=0.029), HER2 positivity (20 [51.3%], P=0.042) and Ki-67 high expression (36 [92.3%], P=0.044) than the non-pCR group. There were significant differences in molecular types between the pCR and non-pCR groups (P<0.001), although luminal B was the main molecular type. The patient characteristics of the tumours in the training and test cohorts are listed in **Table 3**. There was no significant difference in the expression of biomarkers (i.e., ER, PR, HER2, and Ki-67) between the training and test cohorts. Of the 39 patients who achieved pCR at the final pathologic evaluation, 30/91 (32.97%) patients and 9/23 (39.13%) patients achieved pCR in the training and test sets, respectively (**Table 4**).

**Table 2:** Clinical characteristics of pCR and non-pCR breast cancer patients.

Characteristics	non-pCR Group(n= 75)	pCR Group (n= 39)	*P* Value
Age (y)*	50.6 ± 10.7	48.8 ± 11.3	0.367
Max tumour diameter (cm)	3.99 ± 1.49	3.51 ± 1.57	0.112
ER status			<0.001
Negative	12 (16.0)	20 (51.3)	
Positive	63 (84.0)	19 (48.7)	
PR status			0.029
Negative	11 (14.7)	13 (33.3)	
Positive	64 (85.3)	26 (66.7)	
HER2 status			0.042
Negative	52 (69.3)	19 (48.7)	
Positive	23 (30.7)	20 (51.3)	
Ki-67			0.044
Low	19 (25.3)	3 (7.7)	
High	56 (74.7)	36 (92.3)	
Tumour molecular type			0.002
Luminal A	13 (17.3)	1 (2.6)	
Luminal B	54 (72)	25 (64.1)	0.595
HER2 positive	19 (35.2)	11 (44.0)	
HER2 negative	35 (64.8)	14 (56.0)	
HER2 positive (Nonluminal)	3 (4.0)	9 (23.1)	
Triple-negative cancer	5 (6.67)	4 (10.3)	

Data represent the number of patients, and data in parentheses are percentages. *Data are ± standard deviations; ER, oestrogen receptor; PR, progesterone receptor; HER2, human epidermal growth factor receptor 2; Ki-67, antigen Ki67.

**Table 3:** Clinical characteristics of the breast cancer patients in the training and test cohorts.

Variables	Training set (n= 91)	Test set (n= 23)	*P* Value
ER status			1.000
Negative	26 (28.6)	6 (26.1)	
Positive	65 (71.4)	17 (73.9)	
PR status			0.569
Negative	18 (19.8)	6 (26.1)	
Positive	73 (80.2)	17 (73.9)	
HER2 status			0.478
Negative	55 (60.4)	16 (70.0)	
Positive	36 (39.6)	7 (30.4)	
Ki-67			0.381
Low	16 (17.6)	6 (26.1)	
High	75 (82.4)	17 (73.9)	
Tumour molecular type			0.823
Luminal A	10 (11.0)	4 (17.4)	
Luminal B	64 (70.3)	15 (65.2)	0.480
HER2 positive	26 (40.6)	4 (26.7)	
HER2 negative	38 (59.4)	11 (73.3)	
HER2 positive (Nonluminal)	10 (11.0)	2 (8.7)	
Triple-negative cancer	7 (7.7)	2 (8.7)	

Data represent the number of patients, and data in parentheses are percentages. ER, oestrogen receptor; PR, progesterone receptor; HER2, human epidermal growth factor receptor 2; Ki-67, antigen Ki67.

**Table 4:** Clinical characteristics of the training and test sets containing pCR and non-pCR breast cancer patient data.

Variables	Training set (n= 91)		Test set (n= 23)	
	pCR (n=30)	non-pCR (n=61)	pCR (n=9)	non-pCR (n=14)
Age (y)*	50.6 ± 11.3	50.9 ± 11.1	41.9 ± 8.81	49.21 ± 9.13
Max tumour diameter (cm)	3.44 ± 1.46	4.07 ± 1.63	2.71 ± 1.07	2.99 ± 1.38
ER status				
Negative	15 (50.0)	11 (18.0)	5 (55.6)	1 (7.2)
Positive	15 (50.0)	50 (82.0)	4 (44.4)	13 (92.9)
PR status				
Negative	10 (33.3)	8 (13.1)	3 (33.3)	3 (21.4)
Positive	20 (66.7)	53 (86.9)	6 (66.7)	11 (78.6)
HER2 status				
Negative	15 (50)	40 (65.6)	4 (44.4)	12 (85.7)
Positive	15 (50)	21 (34.4)	5 (55.6)	2 (14.3)
Ki-67				
Low	1 (3.3)	15 (24.6)	2 (22.2)	4 (28.6)
High	29 (96.7)	46 (75.4)	7 (77.8)	10 (71.4)
Tumour molecular type				
Luminal A	0 (0)	10 (16.4)	1 (11.1)	3 (21.4)
Luminal B	20 (66.7)	44 (72.1)	5 (55.6)	10 (71.4)
HER2 positive	8 (40.0)	18 (40.9)	3 (60.0)	1 (10.0)
HER2 negative	12 (60.0)	26 (59.1)	2 (40.0)	9 (90.0)
HER2 positive (Nonluminal)	7 (23.3)	3 (4.9)	2 (22.2)	0 (0)
Triple-negative cancer	3 (10.0)	4 (6.6)	1 (11.1)	1 (7.1)

Data represent the number of patients, and data in parentheses are percentages. *Data are ± standard deviations; ER, oestrogen receptor; PR, progesterone receptor; HER2, human epidermal growth factor receptor 2; Ki-67, antigen Ki67.

### Performance Analysis of DBNN Feature Sharing

As mentioned above, it can be understood that the number of layers in a CNN has a specific impact on the prediction and classification performance of the model. Thus, CNNs with different numbers of layers were designed in this experiment. The experimental results were compared to determine the best layer number for the dual branch network. The performance of different convolution layer numbers is shown in the first five rows of **Table 5**. It can be seen that with the deepening of the network, the performance indices of the dual branch model increased first and then decreased in general. Here, X denotes the number of layers of each branch network in CNN-X. CNN-9 performs the best out of the models with different numbers of layers, and it has an accuracy of 81.77%. Moreover, it also ranks the highest in specificity, PPV, and F1-score. Therefore, in this study, the nine-layer CNN was selected as the backbone of the model. Next, the influences of feature sharing and the weight assignment strategy on the model are explored.

**Table 5:** Performance of the model with different convolution layer numbers and feature-sharing methods.

Models	Accuracy (%)	Sensitivity (%)	Specificity (%)	PPV (%)	NPV (%)	F1-score
CNN-8	77.08	69.33	82.05	71.23	80.67	0.703
CNN-9	81.77	69.33	**89.74**	**81.25**	82.03	0.748
CNN-10	76.56	73.33	78.63	68.75	82.14	0.710
CNN-11	77.60	77.33	77.78	69.05	84.26	0.730
CNN-12	75.00	70.67	77.78	67.09	80.53	0.688
CNN-9 FSS	**83.33**	**97.33**	74.36	70.87	**97.75**	**0.820**
CNN-9 FSC	81.77	85.33	79.49	72.73	89.42	0.785

Values in bold black font represent the best performance in each column.

At present, there are many methods of feature sharing, including feature element sum and feature concatenation, which are the classic feature fusion methods (42–46). Thus, we also explored the influence of two different strategies on model performance. In the last two rows of **Table 5**, the performance comparison results of the model with different feature-sharing strategies are shown. CNN-9 FSS represents the CNN model that uses the feature element sum method, while CNN-9 FSC represents the CNN model that uses the feature concatenation method. **Table 5** shows that the model achieves better performance when the feature element sum method is used. The accuracy, sensitivity, NPV, and F1-score values were higher than those obtained by the CNN with feature concatenation and CNN-9 without feature sharing. Therefore, DBNN adopts the feature element sum method as its feature-sharing method.

### Weight Assignment of DBNN Feature Connection

DBNN is a dual-branch network with two inputs and one output, and the two inputs are NAC_pre_ and NAC_1_ chemotherapy data. The output is the probability of predicting pathological results. Therefore, a feature map from each branch network needs to connect the features and then maps from a high-dimensional vector to a low-dimensional vector to complete the classification task. We compared the experimental results of the feature element sum method, feature concatenation method, and feature weight assignment method of the dual-branch network to explore different feature connection methods (see **Table 6**). CNN-9 FSS_concat represents the CNN model with the feature concatenation method, and CNN-9 FSS_sum represents the CNN model with the feature element sum method. CNN-9 FSS (A, B) represents the CNN model with the weight connection method, where A is the weight of the NAC_pre_ branch and B is the weight of the NAC_1_ branch.

**Table 6:** Performance of the model with different feature connection methods.

Models	Accuracy (%)	Sensitivity (%)	Specificity (%)	PPV (%)	NPV (%)	F1-score
CNN-9 FSS_contact	83.33	**97.33**	74.36	70.87	**97.75**	0.820
CNN-9 FSS_sum	82.81	82.67	82.91	75.61	88.18	0.790
CNN-9 FSS (0.9, 0.1)	85.94	82.67	**88.03**	**81.58**	88.79	0.821
CNN-9 FSS (0.8, 0.2)	83.85	85.33	82.91	76.19	89.81	0.805
CNN-9 FSS (0.7, 0.3)	82.81	81.33	83.76	76.25	87.50	0.787
CNN-9 FSS (0.6, 0.4)	77.08	85.33	71.79	65.98	88.42	0.744
CNN-9 FSS (0.5, 0.5)	83.33	78.67	86.32	78.67	86.32	0.787
CNN-9 FSS (0.4, 0.6)	84.38	81.33	86.32	79.22	87.83	0.803
CNN-9 FSS (0.3, 0.7)	82.29	73.33	**88.03**	79.71	83.74	0.764
CNN-9 FSS (0.2, 0.8)	**87.50**	90.67	85.67	80.00	93.46	**0.850**
CNN-9 FSS (0.1, 0.9)	83.85	82.67	84.62	77.50	88.39	0.800

Values in bold black font represent the best performance in each column.

As shown in **Table 6**, when the feature weight of the NAC_pre_ branch is 0.2 and when that of the NAC_1_ branch is 0.8, the model’s performance is the best, with an accuracy of 87.50%. In addition, the F1-score is higher than that of the other models, which may be because NAC_1_ stage data contributed more to the prediction than NAC_pre_ stage data. It can be seen from the last nine rows of **Table 6** that the average accuracy and F1-score values are superior when the NAC_1_ branch is heavier than the NAC_pre_ branch. Therefore, the method of weight connection is adopted in the model, and the experimental results show that this method can achieve the best results. In the following experiments, CNN-9 FSS (0.2, 0.8) is called DBNN.

### Results of DBNN Data Augmentation

As stated earlier, there was a data imbalance problem in RJNAC. The amount of data with non-pCR pathological results was approximately twice that with pCR pathological results, affecting the model’s performance. Therefore, we explored the impact of different data augmentation strategies on the performance of DBNN. The experimental results were compared using nonaugmented data, geometrically transformed data (47), Mixup data (48), and small amounts of upsampled data. Geometric transformation techniques include rotations, flips, and zooming to generate new training samples to maintain realistic tumour shapes. Moreover, small amounts of data upsampling techniques apply geometric transformations to non-pCR examples to achieve a quantity balance between the two categories, solving the data imbalance problem manually.

As seen from **Table 7**, the performance of the model is better without data augmentation. First, it can be seen that the performance of the model on nonaugmented data was better than that of the model on geometrically transformed data. Augmenting both types of data aggravate the data imbalance, leading to degradation in the performance of the model; hence, Mixup data augmentation also degrades model performance. In addition, Mixup may not be suitable for the augmentation of medical datasets because it disturbs the relationship between a lesion and the surrounding area, making the model learn incorrect information. Finally, we enhance the sample size of the two types of data so that they are consistent by sampling small numbers of samples. The experimental results on the augmented data were not as good as the results on the nonaugmented data. Perhaps DBNN learns the redundant features of the data during the learning process, resulting in model performance degradation.

**Table 7:** Performance of DBNN with different data augmentation strategies.

Strategies	Accuracy (%)	Sensitivity (%)	Specificity (%)	PPV (%)	NPV (%)	F1-score
Nonaugmentation	**87.50**	**90.67**	85.67	**80.00**	**93.46**	**0.850**
Geometric transformation	76.04	64.00	83.76	71.64	78.40	0.676
Mixup	70.83	46.67	**86.32**	68.63	71.63	0.556
Small amount of upsampling	79.69	77.33	81.20	72.50	84.82	0.748

Values in bold black font represent the best performance in each column.

### Comparison With the Single Branch Models

**Figure 8:**
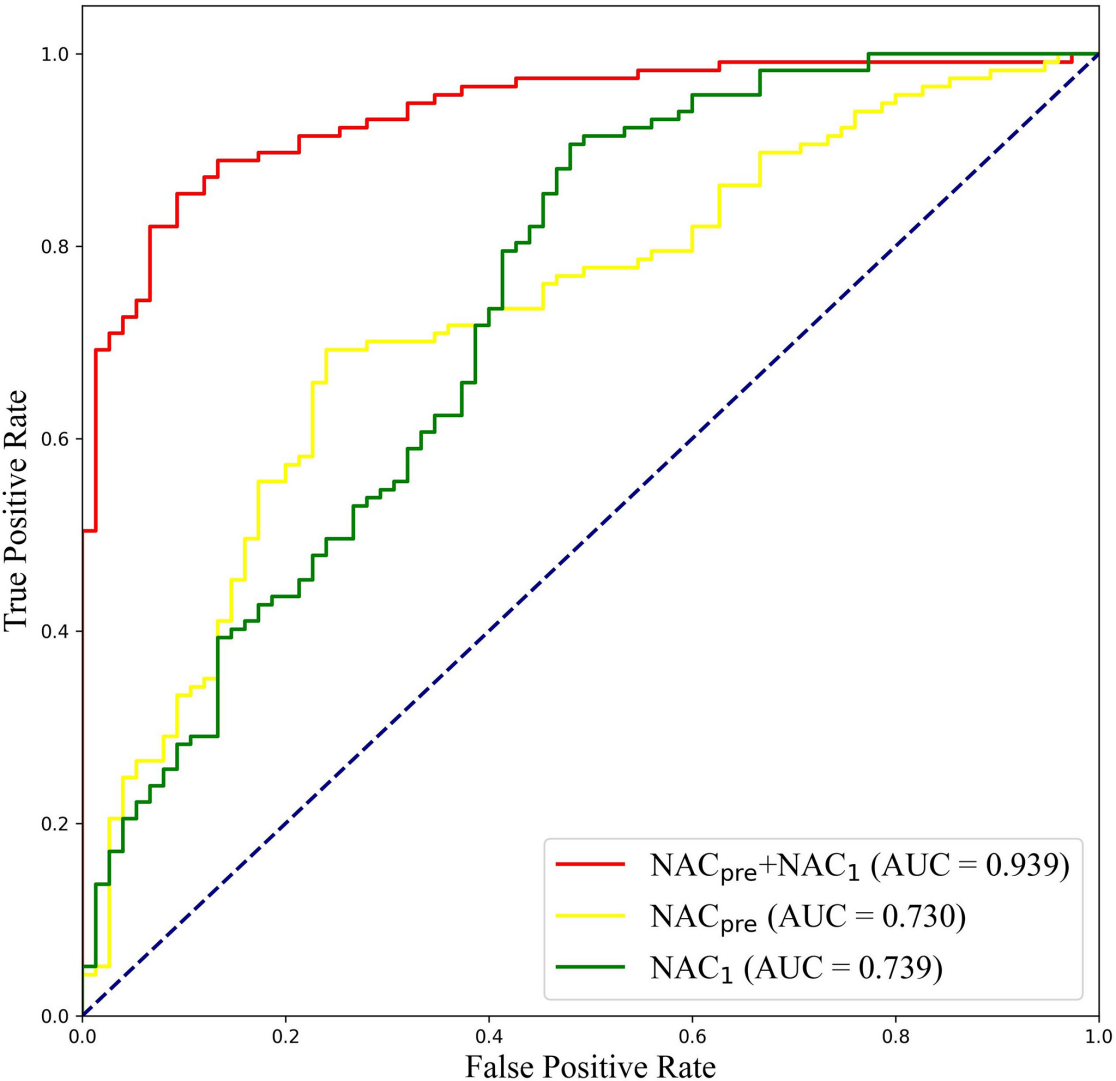
ROC curves of DBNN using data from different chemotherapy stages.

To further validate the potential of DBNN in predicting the efficacy of NAC, it was used to predict the pathological classification of patients early based on the different stage data of NAC treatment in the RJNAC dataset. Compared with the AUC value in the first two rows and the last row in **Table 8**, we know that the model’s prediction results when using a single branch network for single-stage data were not as good as those when using multistage data. In addition, the performance of the model trained on the NAC_1_ data was slightly superior to that trained on the NAC_pre_ data when using single-stage data, which indicates the necessity of DBNN weight assignment. From **Table 8** and **Figure 8**, we can see that the areas under the ROC curve for NAC_pre_ (Az_pre_), NAC_1_ (Az_1_) and NAC_pre_+NAC_1_ (Az_pre+1_) were 0.730, 0.739 and 0.939, respectively. The performance of the model trained on the NAC_1_ data shows higher specificity than that trained on the NAC_pre_ data. The sensitivity of the model trained on NAC_pre_ was superior to that trained on NAC_1_ data. The value of Az_pre+1_ was significantly higher than that of Az_pre_ and Az_1_ (*P <*0.01). However, there was no significant difference between the values of Az_pre_ and Az_1_ (P =0.3244).

**Table 8:** Performance evaluation of DBNN using data from different chemotherapy stages.

Data	Accuracy (%)	Sensitivity (%)	Specificity (%)	PPV (%)	NPV (%)	F1-score	AUC (95% CI)	P value
NAC_pre_	71.35	76.00	68.38	60.64	81.63	0.675	0.730 (0.657,0.802)	<0.01
NAC_1_	73.44	53.33	**86.32**	71.43	74.26	0.611	0.739 (0.664,0.813)	<0.01
NAC_pre_+NAC_1_	**87.50**	**90.67**	85.67	**80.00**	**93.46**	**0.850**	**0.939** (0.907,0.972)	**-**

Values in bold black font represent the best performance in each column.

Moreover, some more sophisticated deep learning models were tested for single branch classification due to CNN-9 was used to train single branch models. The experimental results are shown in **Table 9**. The AUC of CNN-9 on NAC_pre_ data was the highest (AUC=0.730), and the AUC value on NAC_1_ data was very close to the optimal value (0.739 vs. 0.756). Therefore, we believe that CNN-9 can be used as a representative of the classical single branch network.

**Table 9:** Comparison of CNN-9 and sophisticated DL models for single branch classification.

Methods	Data	Accuracy (%)	Sensitivity (%)	Specificity (%)	PPV (%)	NPV (%)	F1-score	AUC
CNN-9	NAC_pre_	71.35	**76.00**	68.38	60.64	**81.63**	**0.675**	**0.730**
	NAC_1_	73.44	53.33	86.32	71.43	74.26	0.611	0.739
ResNet	NAC_pre_	70.83	60.00	77.78	63.38	75.21	0.616	0.681
	NAC_1_	73.44	42.67	93.16	80.00	71.71	0.557	0.748
EfficientNet	NAC_pre_	67.19	70.67	64.96	56.38	77.55	0.627	0.710
	NAC_1_	72.92	**74.67**	71.79	62.92	**81.55**	**0.683**	0.750
MobileNet	NAC_pre_	**72.92**	60.00	**81.20**	**67.16**	76.00	0.634	0.711
	NAC_1_	**73.96**	46.67	91.45	77.78	72.79	0.583	**0.756**
ResNeXt	NAC_pre_	69.79	53.33	80.34	63.49	72.87	0.580	0.653
	NAC_1_	71.35	33.33	**95.73**	**83.33**	69.14	0.476	0.639
ShuffleNet	NAC_pre_	66.15	42.67	81.20	59.26	68.84	0.496	0.640
	NAC_1_	71.35	66.67	74.36	62.50	77.68	0.645	0.707
WRN	NAC_pre_	67.71	58.67	73.50	58.67	73.50	0.587	0.668
	NAC_1_	72.92	57.33	82.91	68.25	75.19	0.623	0.726

Values in bold black font represent the best performance in each column.

### Comparison With the Latest Studies

**Figure 9:**
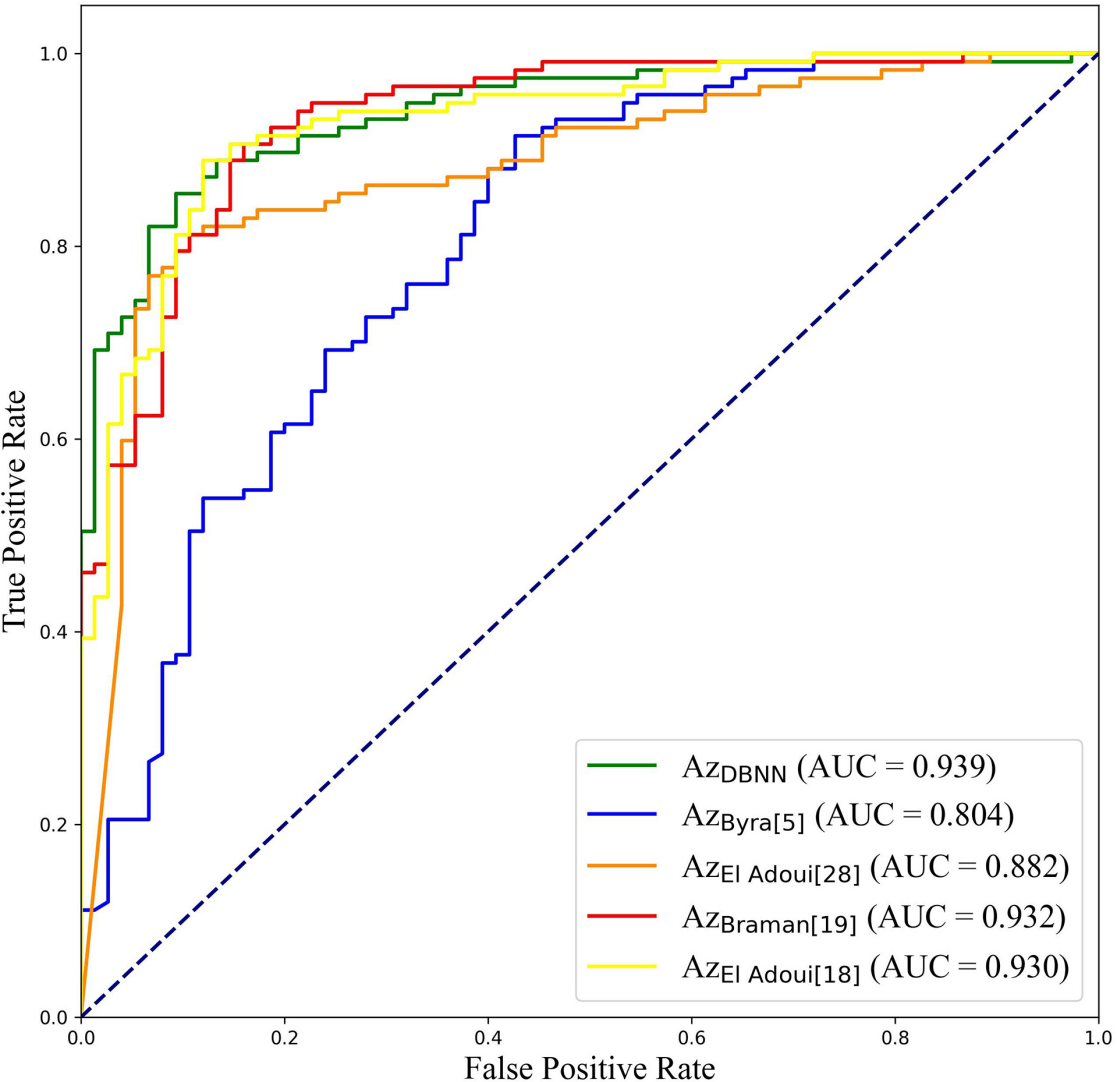
ROC curves of DBNN and other NAC prediction methods.

**Figure 10:**
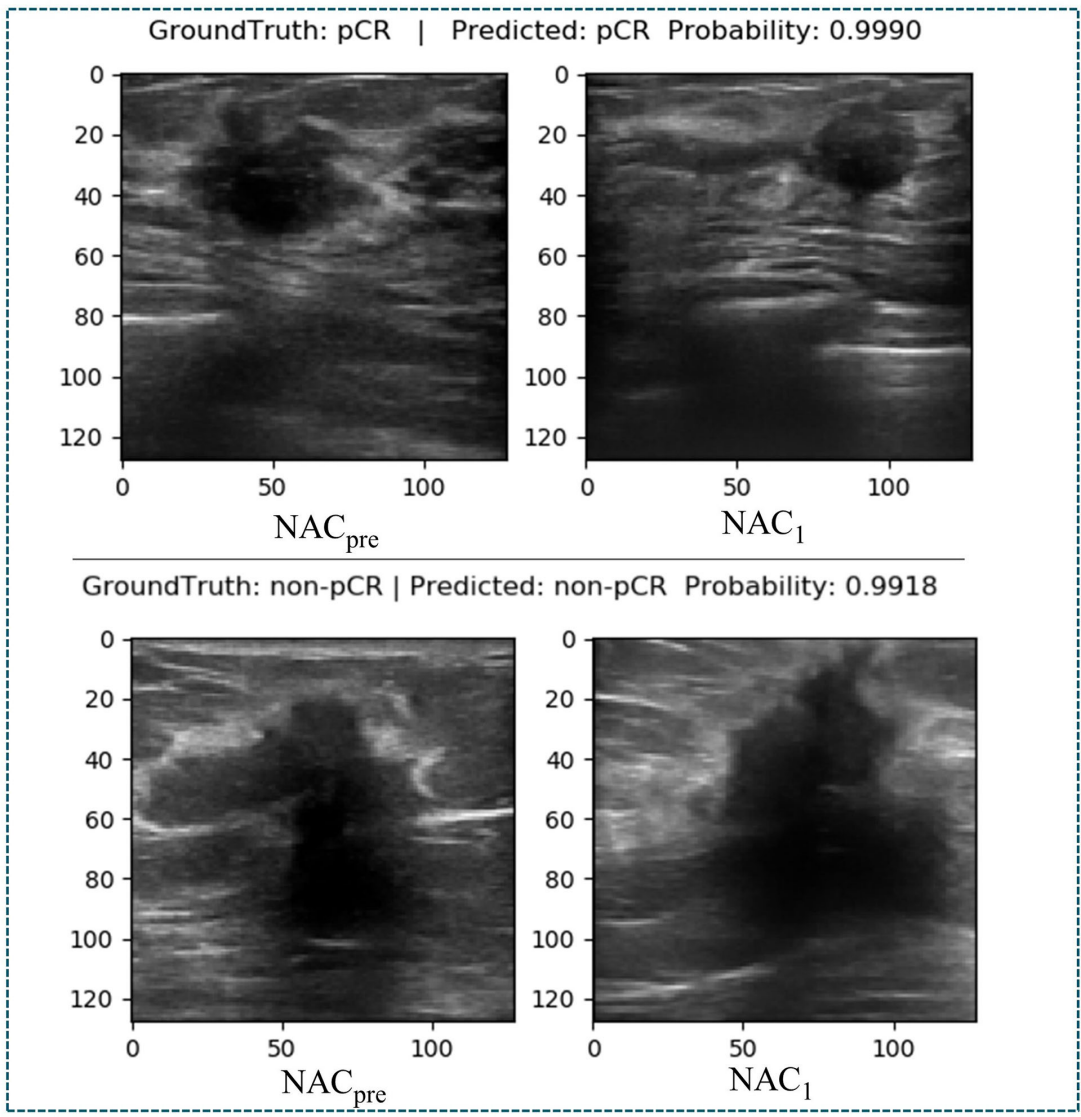
Diagram of the ground truth and prediction results.

At present, there are few studies on the prediction of NAC response for breast cancer based on US images, and the datasets used in each study and each imaging protocol are different, so it is difficult to compare the results directly. However, to verify the research value of DBNN, this study referred to the four latest papers, reproduced the methods according to the technical details described in the articles, and applied them to the RJNAC dataset (5, 18, 19, 28). Two identical Inception-ResNet-V2 CNNs based Siamese models without fine-tuning were reimplemented to extract generic features. Then the difference between the feature vectors was used to train a logistic regression model for the prediction (5). We reimplemented a two-input CNN, in which each input branch consisted of four blocks of 2D convolution layers, each followed by a ReLU activation function and max-pooling layer. A dropout layer was applied after every two convolutional blocks. Then, the two branches were concatenated after a fully connected layer followed by ReLU, dropout (with a rate of 40%), and a Sigmoid function for the final classification (18), while two dense layers were processed to yield the final output (19). The developed multi-input deep learning architecture contained two parallel sub-architectures with similar layers to the single architecture, consisting of six blocks with multiple convolutional layers, each followed by a ReLU activation function and max-pooling layer. Then, a concatenation was applied between two single architectures, a dropout of 50%, and a fully connected layer was used at the end of the network to provide a classification result (28). In these studies, three of the approaches were based on MRI data (18, 19, 28), and one was based on US image data (5). In **Figure 9**, the area under the ROC curve for DBNN (Az_DBNN_) was significantly higher than that of Az_Byra_ (5) (P =0.004). However, there was no significant difference in the values of Az_DBNN_ and the values of the area under the ROC curve for the other methods (**Table 10**). We also show the prediction results and pathology labels of the model on NAC_pre_ and NAC_1_ images and the probability of the model output prediction results in **Figure 10**.

**Table 10:** Comparison of DBNN and other NAC prediction methods on the RJNAC dataset.

Methods	Accuracy (%)	Sensitivity (%)	Specificity (%)	PPV (%)	NPV (%)	F1-score	AUC (95% CI)	P value
DBNN	**87.50**	**90.67**	85.67	80.00	**93.46**	**0.850**	**0.939 **(0.907,0.972)	**-**
Byra (5)	77.08	52.00	**93.16**	82.98	75.17	0.639	0.804 (0.739,0.869)	0.004
El Adoui (28)	82.81	81.33	83.76	76.25	87.50	0.787	0.882 (0.830,0.930)	0.099
Braman (19)	**87.50**	82.67	90.60	84.93	89.08	0.838	0.932 (0.897,0.968)	0.500
El Adoui (18)	86.46	76.00	**93.16**	**87.69**	85.83	0.814	0.930 (0.894,0.966)	0.381

Values in bold black font represent the best performance in each column.

## Discussion

The early prediction of chemotherapy response in patients with breast cancer is crucial for improving and personalizing patient treatment. In this study, a novel deep learning method, DBNN, based on US images for the early prediction of NAC response in patients with breast cancer was proposed and validated. The experimental results showed that the best prediction performance was obtained with the DBNN model using feature sharing and weight assignment. It was worth noting that all the performances shown in **Tables 5**–**10** were from the test set. The highest diagnostic performance was obtained when the US image information of NAC_pre_ and NAC_1_ was combined, in which the accuracy, sensitivity, specificity, F1-score, and AUC values were 87.50%, 90.67%, 85.67%, 0.850, and 0.939, respectively.

The DBNN approach for the early prediction of NAC proposed in this study has several advantages.

First, compared to the previous traditional machine learning methods, which mainly depend on feature engineering and require domain knowledge to build feature extractors, our deep learning approach is automatic and does not require feature engineering. Methods based on machine learning are limited in their function, as they are dependent on handcrafted features. Moreover, our model considers not only the tumoral region but also the tumour’s surrounding tissue by using entire breast tumour images. Supplementing the US features extracted from a tumour itself with features computed within the tumour’s surrounding tissue, such as the peritumoural region, may improve the prediction of pCR from US images (49, 50). Second, different from the existing deep learning algorithms, DBNN fuses features of each branch in the process of extracting low-level features, which may effectively screen out important features through the training network to achieve more accurate early prediction results. Third, in contrast to the existing methods for predicting NAC response using two-stage data, we assume that the importance of the data before and after chemotherapy is inconsistent. Therefore, DBNN introduces the weight assignment strategy to increase the weight of data features after chemotherapy by using prior knowledge to guide network training to affect the NAC response prediction results.

It is difficult to directly compare our results to those of other methods reported in other studies due to different data acquisition techniques, analysis protocols and subject groups. Moreover, there are few studies that use deep learning for NAC response early prediction in breast cancer based on US images. Nevertheless, we can compare our results with those of models trained on our datasets. The studies performed by El Adoui et al. (28), Braman et al. (19), and El Adoui et al. (18) were based on MRI data, and the study designed by Byra et al. (5) was based on US image data. All four methods are two-input CNN architectures for the prediction of breast tumour NAC response from follow-up images. Each branch was operated on by a series of convolution-based operations and summarized into a set of deep features, which were then combined and processed by the feature fusion of two branches to generate a final score representing the response probability. However, those methods only considered the late fusion of deep features. The models cannot effectively share data features at different stages in their respective branches and may even filter out crucial features, such as changes in lesion areas. Therefore, they cannot make full use of the relationship between different data for model training. In **Table 10**, comparisons of the performance of the state-of-the-art methods and our method were made based on seven indices: accuracy, sensitivity, specificity, PPV, NPV, F1-score and AUC. Our method obtained better results on most of the evaluation indices. The ROC curves based on the true positive rate (TPR) and false positive rate (FPR) for the existing methods and our proposed method are shown in **Figure 9**. The AUC values of all the algorithms were over 0.8, and the largest AUC value (0.939) was obtained by our model. The area under the ROC curve obtained by DBNN (Az_DBNN_) was significantly higher than that obtained by Az_Byra_ (5) (P =0.004). The model developed by Byra et al. (5) was based on a small dataset with images from 30 patients, while our dataset contained images from 114 patients. We can train our deep learning model from scratch because a model pretrained on natural images is often not the best model when applied to medical images. Moreover, we shared the data features of the two streams in the training process and assigned the weights of the different stages by using prior knowledge to obtain more accurate results.

Although the proposed method has improved the prediction accuracy of NAC response, there are still some limitations in this study. First, due to the small dataset of US images collected from a single centre, the model’s generalization ability needs to be further improved. Since there is currently no public dataset of ultrasound images before and after the first stage of chemotherapy for NAC, our next work will continue to collect data from multi-centres to further verify our model’s generalization ability. It is generally accepted that the larger a dataset is, the better the performance of the deep learning models (51, 52). Limited datasets are a prevalent challenge in medical image analysis. Second, due to the heterogeneous nature of the histopathologic and molecular subtypes of breast cancer included in our study, the pathologic response to NAC may be affected and may cause selection bias. Finally, we did not add breast cancer molecular subtype to our method, which may help to predict the response of breast cancer to NAC early. The application of DBNN is only in the primary stage. Therefore, how to extend our method to clinical decision-making is worthy of in-depth study.

In the future, there will be at least two aspects of NAC response prediction models based on different stages of data that can be further developed. On the one hand, DBNN should also consider more feature methods, such as combining low-level features and high-level features by utilizing residual cross-branch connections. Moreover, adaptive weight allocation can be regarded as the weight assignment strategy. On the other hand, the robustness and generalization ability of DBNN need further verification.

In conclusion, our study proposes a novel dual-branch DBNN model based on feature sharing and weight assignment to predict the efficacy of NAC treatment for breast cancer utilizing greyscale US images. DBNN has two remarkable advantages: feature sharing and weight assignment. Feature sharing can make the model consider the correlations between data in different stages of NAC during training. Moreover, weight assignment, which provided prior knowledge, emphasizes the importance of data at different NAC treatment stages. The results show that DBNN has the potential to enable the early prediction of pCR and achieved good prediction performance when applied on NAC_pre_ and NAC_1_ data. However, a further large-scale study with an independent external validation dataset is needed before this approach can be used for actual clinical decision-making, and it may become an important monitoring tool for the early prediction of the response to NAC in patients with breast cancer.

## Data Availability Statement

The data analyzed in this study is subject to the following licenses/restrictions: Due to the privacy of patients, the related data cannot be available for public access. Requests to access these datasets should be directed to Caifeng Wan, wancaifengky@sina.com.

## Ethics Statement

The studies involving human participants were reviewed and approved by Shanghai Jiao Tong University School of Medicine, Ren Ji Hospital Ethics Committee. Written informed consent was waived in this study.

## Author Contributions

Conception and design: JX, CD, and XS. Collection and assembly of data: CW, QD and CD. Verification of the underlying data: HS, XS, and JW. Development of methodology: JX, HS, XS, and JW. Data analysis and interpretation: JX, HS, CW, XS, QD, and CD. Writing original draft: JX, HS, CW, QD, CD, and JW. All authors contributed to the article and approved the submitted version.

## Funding

This work was supported by National Natural Science Foundation of China (Grant No.61873156, Grant No. 81801697, Grant No. 81571678).

## Conflict of Interest

The authors declare that the research was conducted in the absence of any commercial or financial relationships that could be construed as a potential conflict of interest.

## Publisher’s Note

All claims expressed in this article are solely those of the authors and do not necessarily represent those of their affiliated organizations, or those of the publisher, the editors and the reviewers. Any product that may be evaluated in this article, or claim that may be made by its manufacturer, is not guaranteed or endorsed by the publisher.
